# Tools and frameworks for evaluating the implementation of learning health systems: a scoping review

**DOI:** 10.1186/s12961-024-01179-7

**Published:** 2024-08-06

**Authors:** Darren Rajit, Sandra Reeder, Alison Johnson, Joanne Enticott, Helena Teede

**Affiliations:** 1 Monash Centre for Health Research and Implementation, Faculty of Medicine, Nursing, and Health Sciences, Monash University, Melbourne, VIC Australia; 2 Monash Partners Academic Health Sciences Centre, Melbourne, VIC Australia; 3 Monash Health Endocrinology and Diabetes Departments, Melbourne, VIC Australia

**Keywords:** Learning health systems, Evaluation, Implementation science, Healthcare improvement

## Abstract

**Introduction:**

Despite increased interest in learning health systems (LHS), a paucity of guidance and tools for evaluating LHS implementation exists. To address this, we aim to undertake a scoping review on existing tools and evaluation of exemplars of LHS implementation.

**Methods:**

We conducted a scoping review of peer-reviewed studies within Scopus, EMBASE, MEDLINE, and MEDLINE in-process that described (1) the evaluation of the implementation of an operating LHS or (2) the development of a framework or tool to facilitate this evaluation. Anima, basic research, abstracts, non-English language articles, and publications before 2018 were excluded. All study designs were considered.

**Findings:**

From 1300 studies initially identified, 4 were eligible, revealing three tools with nine implementation evaluation examples. The identified tools shared constructs which were evaluated, including: Stakeholders, Data, Research Evidence, Implementation, and Sociotechnical Infrastructure. However, there was divergence in evaluation methodology. Tools ranged from a five-point numerical rating system for process maturity with a radar chart called the Network Maturity Grid (NMG); the Kaiser Permanente Washington (KPWA) LHS Logic Model, which provides a broad list of constructs and sample measures relevant to LHS operations; and finally LADDERS, a simple tool or form-based template designed for consistent evaluation over time. The NMG tool was the most mature in terms of adaptation and adoption. Notably, two (NMG and the KPWA LHS Logic Model) out of three tools conceptualized the LHS as a suite of processes and devised tools were processes that linked these constructs.

**Implications for toolkit development:**

The evaluation of LHS implementation remains an under explored area of investigation, as this scoping review found only three tools for LHS implementation evaluation. Our findings indicate a need for further empirical research in this area and suggest early consensus in constructs that need to be considered during evaluation.

## Introduction

Learning health systems (LHS), conceptualized initially by the US Institute of Medicine, embody an aspirational vision for health systems where people, technology, and culture are seamlessly integrated to facilitate cyclical and systemic healthcare improvement [1]. Proposed in the early 2000s to tackle the challenges of integrating evidence-based medicine (EBM) at scale and to leverage the potential of big data and electronic health records (EHR), the LHS has seen emerging international adoption across countries such as the USA, Australia, Canada, and Sweden [2].

Central to the LHS is the concept of “learning,” which involves using evidence gathered from various stakeholders, including patients and clinicians, and data generated as patients journey through the health system, informing care and decision-making in near real-time. In the LHS model, “evidence” extends beyond evidence from traditional research to evidence from stakeholder experience and priorities, evidence from research such as randomized controlled trials and systematic reviews to encompass evidence from practice focused on data, and evidence from implementation in the local setting. Examples include prioritizing consumer involvement through the collection of patient-reported outcomes (PROMs) and experience measures (PREMs) [3, 4], leveraging EHR data [5, 6], and integrating implementation science strategies into healthcare improvement [7, 8].

The application of the LHS across a variety of health systems and settings has demonstrated positive impact [2]; ranging from improvements in (1) evidence-based guideline compliance in lung cancer care [9], despair and distress measures in patients with breast cancer [10], and patient visit communication and glycemic control among people with type 2 diabetes [11]. However, despite increased adoption and evidence for impact, much of the LHS literature remains theoretical [12], and the emerging empirical evidence remains limited [13], with exemplars being confined to local contexts or singular clinical settings [13]. As a result, it is yet unclear what aspects of a LHS directly leads to improved outcomes, limiting continued adoption and sustained scale-up.

There is also a corresponding lack of LHS-specific tools that can evaluate how well an existing implementation of a LHS aligns with the objectives and vision. Such tools are important in allowing robust and comparable evaluations of the impact of different LHSs in variable contexts. Due to the diverse and at times theoretical nature of the LHS, we conduct a scoping review to address this knowledge gap by identifying and summarizing the existing research on the evaluation of existing LHS implementation. We plan to capture such tools, alongside examples of the use of such tools, identify residual gaps, and capture opportunities for further research. Ultimately, this review aims to inform ongoing development of a pragmatic, evidence-based toolkit to support ongoing real-world implementation and evaluation LHS efforts to bridge the conceptual gap between theory and practice in the LHS.

### Aim

The aim of this scoping review is to gather evidence on the evaluation of existing LHS implementation and report on current development and use of LHS specific evaluation methodologies, tools and frameworks.

### Context

**Fig. 1:**
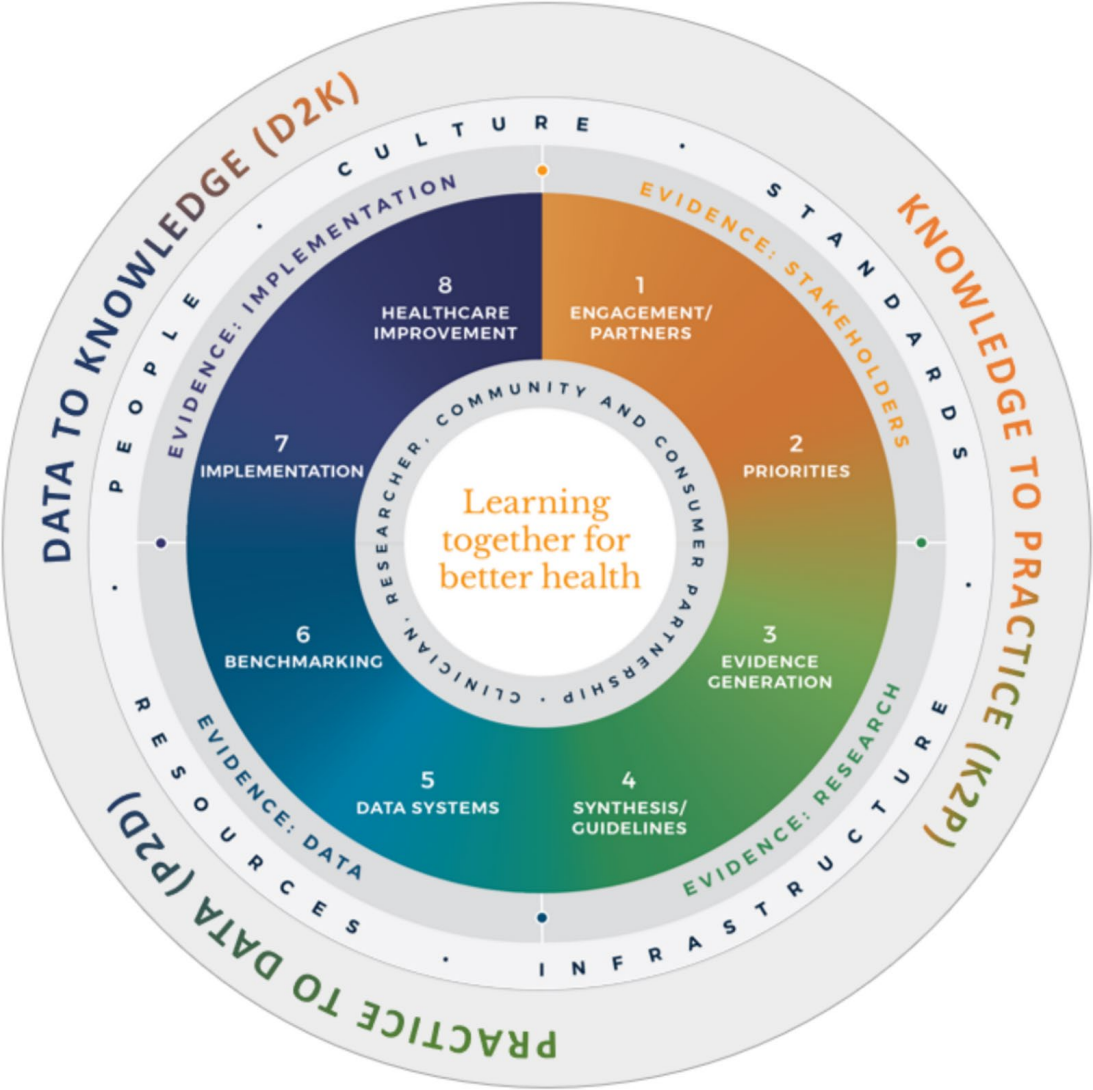
Monash LHS framework

This scoping review was conducted within the context of an Academic Health Centre (https://mchri.org.au/) that is working with existing health services to implement a rigorously codesigned Monash LHS model [14] (Fig. 1). The Monash LHS model [14] was developed through a combination of stakeholder driven codesign, systematic literature review [2], qualitative research, and consensus processes.

It highlights key evidence sources that must be considered to continuously translate knowledge into practice, that is evidence from stakeholders, research, data and implementation (as illustrated in the four main quadrants in Fig. 1). It further integrates core phases of evidence generation and synthesis across all four evidence types, ranging from stakeholder engagement and priority setting related to stakeholder evidence, primary evidence generation and guideline development related to research evidence, data systems and benchmarking relevant to data evidence, and the application of implementation science and existing healthcare improvement methodologies relevant to implementation evidence. As the framework diverged from existing LHS frameworks due to adopting a systems level lens and integrating a stakeholder-centered perspective, the model has found increased adoption within the Australian context, implementation work underway.

However, while implementing the LHS, stakeholders had identified the need for tools and frameworks usable by front line implementation researchers and health service teams to evaluate ongoing implementation and to support alignment with the Monash LHS model. A recent scoping review of LHS literature [13] conducted in 2021 had found very limited research in the production of LHS specific tools, with existing examples focused on either adapting existing frameworks such as the Consolidated Framework of Implementation Research (CFIR) for use within an existing LHS [15]. The review also noted a dearth in high quality empirical research, including implementation evaluation studies [13].

Thus, the authors, which include a researcher in residence (D.R.), health service manager (A.J.), and LHS experts (H.T., A.J., and J.E.) aim to realize the stated aims and results of the scoping review to inform the ongoing coproduction of an implementation evaluation toolkit for integration into existing LHS activities. Thus, the Monash LHS framework’s key evidence sources of stakeholders, research, data, and implementation is used as a basis for synthesis in this scoping review. This is to ensure proper alignment with ongoing, government funded efforts to implement the Monash LHS framework and support stakeholder uptake [16]. Further, this scoping review is the continuation of work on the impact of the LHS globally [2]. Aligned to this rapidly emerging field, the search in this scoping review was conducted from 2018 onwards.

## Methods

### Search strategy

The scoping review was conducted according to guidance from Arskey et al. [17] and Levac et al. [18] and the Preferred Reporting Items for Systematic Reviews and Meta-Analyses (PRISMA-ScR) guidelines for scoping reviews [19]. A protocol was developed prospectively and is available on request. An electronic search was conducted within Scopus, EMBASE, MEDLINE, and MEDLINE in-process in May 2023, utilizing keywords as listed in Appendix 1. Publications were limited to the English language and published within the past 5 years (2018–current). Records were entered into EndNote for deduplication, prior to commencement of title and abstract screening.

### Inclusion and exclusion criteria

Publications that described a (1) example evaluating the implementation of an operating LHS or (2) the development of a framework or tool to facilitate this evaluation were included. Only examples that self-identified as a “learning health system,” “learning health network,” or related variant were included. Additionally, tools and frameworks were defined as any guide, framework, assessment tool, evaluation tool, or rubric, either presented in digital format or otherwise, that can be used to (1) evaluate the maturity or progress of LHS implementation or (2) evaluate how well ongoing health system activities currently align with a particular LHS model. All primary study types were considered, including mixed methods studies, qualitative studies, case studies, and quantitative studies.

Publications that described the implementation of a LHS without describing an evaluation of the implementation process were excluded. Publications that presented a conceptual framework describing a LHS without outlining a process for assessing the implementation of a LHS were also excluded. Animal research, conference and poster abstracts, basic research, non-English language articles, and publications before 2018 were also excluded. All study designs were considered.

### Screening

Title and abstracts of retrieved publications were screened independently by a single author (A.J.) to identify publications that met the inclusion criteria. Full text was then retrieved and independently assessed by two authors (A.J. and D.R.). Shared understanding and consensus for articles eligible for inclusion was generated via regular meetings between A.J. and D.R.

### Data extraction

The first author (D.R.) developed a data extraction template incorporating end user (A.J. and H.T.) input and constructs from the Monash LHS model to ensure better usability for the proposed context. Thus, for publications that described a tool or framework, the (1) aim of the tool, (2) evaluation constructs described in the tool, (3) scoring/evaluation system employed by the tool, and (4) the development methodology of the tool was extracted. The development methodology was further divided into three subthemes in partial alignment with the Monash LHS model (Fig. 1), that is, whether (1) evidence from stakeholders was leveraged through the process, (2) whether the development of tool had leveraged evidence from research/or was theory driven, and (3) whether evidence from implementation into practice had been leveraged, through evidence of piloting in an existing LHS or network.

For publications that described a case study evaluating the implementation of the LHS, the (1) name, (2) country, (3) scale, (4) study design, (5) implementation evaluation tool/framework that was employed, (6) areas of evaluation, (7) users of the tool, (8) reported outcomes of the LHS, and (9) how the results of evaluation were used were extracted.

Data extraction was conducted by one author (D.R.), with four authors (D.R., A.J., J.E., and H.T.) periodically meeting throughout the data extraction process to generate consensus.

## Results

**Fig. 2:**
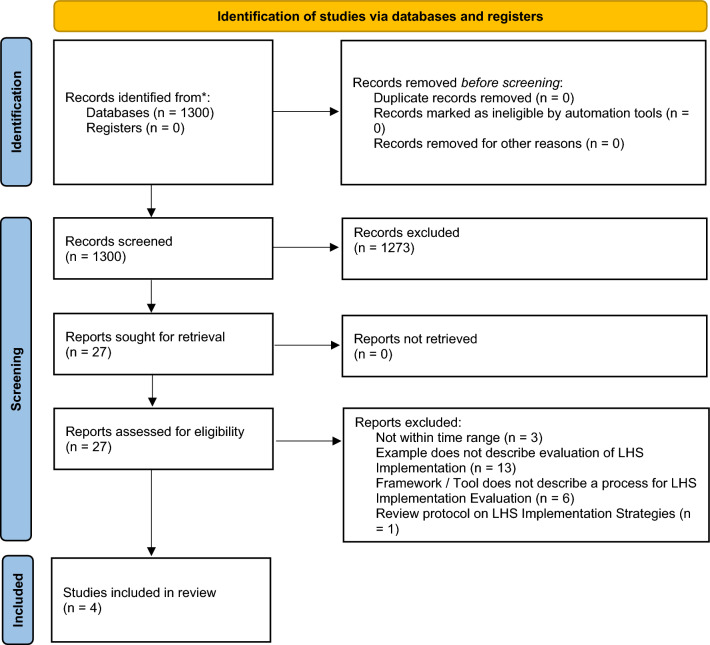
PRISMA diagram

As in Fig. 2, the final search yielded a total of 1300 unique records after deduplication. A total of 1273 records were excluded during the title and abstract screening phase, resulting in 27 records that were sought for full text retrieval and full text screening. A total of 23 records were excluded during full text screening, most frequently due to not describing a process for LHS implementation evaluation (*n* = 19), either for records that described a record or tool (*n* = 6) or a case study (*n* = 13). A total of four full text records [20–23] proceeded to final data extraction, consisting of three tools or frameworks (Table 1), with nine examples (Table 3), eight of which were extracted from Lannon et al. [20] and one from Bailes et al. [23]; although, all nine were conducted with the same tool [20].

**Table 1:** Tools and frameworks eligible for inclusion

Tool name	Aim of tool	Evaluation components/constructs described in tool	Scoring/evaluation System
Network Maturity Grid (NMG*) [20]	To self-assess the maturity of processes across a range of social and technical domains related to achieving the LHS vision	Six domains with associated components: systems of leadership, governance and management, quality improvement, engagement and community building, data and analytics, science	A five‐point numerical rating scale is applied to rate each construct. The scale represents a range of maturity: Category 1: not started, Category 2: beginning Category 3: intermediate Category 4: mature Category 5: idealized state The result is then plotted as a radar chart across the six assessed domains
KPWA LHS logic model*	To provide a broad list of constructs relevant to LHS programs, depict their relationship to LHS operations, harmonizes terms across models, and provide measures for the operationalization of each construct. The model identifies essential LHS inputs, provides transparency into LHS activities, and defines key outcomes to evaluate LHS processes and impact	Inputs [6]: people and partnership, health information infrastructure, prioritization, funding, improvement infrastructure, ethics, and oversight	Non-numerical rating system with prompting questions and sample measures to evaluate progress against all 24 constructs
		Outputs [9]: environmental scanning, evidence synthesis and translation, data analytics, design, patient and family engagement, implementation support, evaluation, dissemination, and consultation	
		Outcomes [9]: knowledge to action latency, systematic implementation of EBP, systematic elimination of wasteful and ineffective practices, population health, care experience, utilization/cost of care, work life for care teams, equity, and programmatic return on investment	
LADDERS	Provides a list of constructs and a template to be considered when organizing and assessing progress over time in planning, implementing, evaluating, and sustaining change at multiple levels within a system	Leadership, alignment, data, demonstration, evaluation, replication, and sustainability	Composed of a brief, three-column template consisting of each ladders element, with corresponding cells for an assessment for each element, and a plan going forwards as a result of this assessment

*High level components have been extracted; however, subcategories have been omitted

### Tools/frameworks identified

Secondly the Kaiser Permanente Washington (KPWA) LHS Logic Model developed by Allen et al. [21], which presents 24 constructs that are relevant to LHS programs. The constructs are divided into 6 inputs, 9 outputs, and 9 outcomes, and a measurable operationalisation of each construct is provided. For example, the “people and partnerships” construct is presented alongside a recommended level of analysis (“Organisation or setting”), suggestions for measurement (through observations and checklists), and sample measures or question prompts (eg: “Does your team have key stakeholder relationships in place to succeed?”). However, a numerical rating system or rubric is not provided. The model was developed through a narrative review of LHS models, components, and measurement approaches.

Our search identified three tools or frameworks (Table 1) that were eligible for inclusion. First, the Network Maturity Grid (NMG) developed by Lannon et al. [20], which is a process-oriented framework designed to assess the maturity of processes within a LHS across six domains utilizing a five point numerical rating system. Each domain consists of eight to nine components, and ratings are delivered on the basis of a self-assessment on the average process maturity (scored 1–5) across all components for a given domain. The results are then plotted as a radar chart. Each component, thus, has a specific process outcome for each maturity level. For example, under the “Expectation that parents, patients, clinicians and researchers are co-creators and co-producers” component of the “Engagement and Community Building” domain, a LHS with “no expectation of collaboration/partnership” would be assessed as a 1 (“not started”) on the maturity scale, whereas a LHS where: “ time and contributions of all partners are valued, demonstrated, celebrated, and acknowledged in fair financial compensation, as well as reasonable and thoughtful request for time commitment” is assessed as a 5 (“idealized state”) on the maturity scale. The model itself was developed through stakeholder input from seven experts in network design and management to identify and refine the evaluated domains, informed by a literature review to identify network organizational tools and major processes associated with LHSs, and was codeveloped and piloted with nine learning networks over 3 years from 2017 to 2019.

Lastly, a tool developed by Meissner. [22], which presents the constructs commonly cited by health systems that have undergone successful transformational change, under the acronym “LADDERS”, that is, Leadership, Alignment, Data, Demonstration, Evaluation, Replication, and Sustainability. The tool is presented as a template with a column for each LADDER construct, a column for an assessment of the current state for each LADDER construct, and lastly a column for a plan stemming from the prior assessment for each LADDER construct. The tool is recommended for use as a documentation tool to capture health systems learning over time and was developed through a synthesis of the author’s experiences over time.

Notably, some convergence in the LHS constructs that were evaluated by these tools were noted. These tools reflected underlying consensus on key constructs that contribute to the successful implementation and sustainability of a LHS. Constructs could be categorized into processes or outcomes across five broad themes: stakeholders, research evidence, data, implementation, and finally, the underlying sociotechnical infrastructure required to support these processes and outcomes, as depicted in Table 2.

**Table 2:** Constructs described in each tool mapped to themes: stakeholders, research evidence, data, implementation, and sociotechnical infrastructure

Tool name	Stakeholders	Research evidence	Data	Implementation	Sociotechnical infrastructure
Network Maturity Grid* [20]	Engagement and community building	Science	Data and Analytics	Quality improvement	Systems of leadership, governance, and management
KPWA LHS Logic Model*	People and partnership, prioritization, patient and family engagement, consultation, work life for care teams, care experience	Evidence synthesis and translation, systematic implementation of EBP, population health,	Data analytics	Design, implementation support, evaluation, dissemination, consultation, knowledge to action latency, programmatic return on investment, utilization/cost of care	Health information infrastructure, funding, improvement infrastructure, ethics and oversight, environmental scanning, equity
LADDERS	Alignment		Data	Demonstration, evaluation, replication, and sustainability	Leadership

*High level components have been extracted; however, subcategories have been omitted

However, divergence in terms of implementation evaluation methodologies were observed (Table 3). The NMG [20] is the most prescriptive of the identified tools, with its five-point numerical rating system for process maturity, and the provision of a radar chart to allow at-a-glance assessment of areas for improvement within a LHS. This is followed by the KPWA LHS Logic Model [21], which provides a broad list of constructs, level of analysis (organization versus setting levels) at which evaluation can be taken and sample measures relevant to LHS operations, but it stops short of offering an off-the-shelf tool for evaluation. Lastly, the LADDERS tool [22], which is the simplest and most flexible of the eligible tools, consists simply of a form-based template designed for consistent use over time.

**Table 3:** Development methodology of the identified tools

Tool name	Development methodology		
	Was evidence from stakeholders leveraged?	Was evidence from research leveraged? (i.e., theory driven)	Was evidence from implementation leveraged (i.e., piloted) and further refined?
Network Maturity Grid			
KPWA LHS Logic Model			
LADDERS			

Notably, only the NMG was developed through leveraging evidence from stakeholders, research, and implementation as in Table 3 and represents the tool that is most mature in terms of adaptation and adoption. It has already been piloted over 3 years in its initial development and is already in use in established research illustrated in Table 4.

**Table 4:** Identified LHS implementation evaluation case studies (all originating from the USA)

LHS name	Years since established at time of evaluation	LHS scale
ImproveCareNow Network	10	Regional
Ohio Perinatal Quality Collaborative	9	Regional
Solutions for patient safety	8	Regional
National Pediatric Cardiology Quality Improvement Collaborative	8	Regional
Autism Speaks: Autism Treatment Network/Autism Intervention Research Network on Physical Health	2	Regional
Cystic Fibrosis Learning Network	2	Regional
Improving Renal Outcomes Collaborative	1	Regional
All Children Thrive Learning Network	2	Regional
Cerebral Palsy Research Network	5	Regional

### Implementation evaluation examples identified

Our search identified nine implementation evaluation examples (Table 4) of existing LHS implementations, all utilizing the NMG tool (Table 5) [3]. These examples focused on LHSs in the USA at a regional scale, with implementation evaluations conducted on LHSs that were established on average, 5.2 years before the start of evaluation.

**Table 5:** Details of LHS implementation evaluation case studies (all conducted with the Network Maturity Grid (NMG) tool)

LHS name	Areas of evaluation	Stakeholders approached during evaluation	LHS outcomes	How results of evaluation was used
ImproveCareNow Network	Across the six domains of the Network Maturity Grid model (Systems of Leadership, Governance and Management, Quality Improvement, Engagement and Community Building, Data and Analytics, and Research.)	Learning network leaders**	Not reported	Informed Evaluation Tool development
Ohio Perinatal Quality Collaborative	Across the six domains of the Network Maturity Grid model (Systems of Leadership, Governance and Management, Quality Improvement, Engagement and Community Building, Data and Analytics, and Research.)	Learning network leaders**	Not reported	Informed Evaluation Tool development
Solutions for patient safety	Across the six domains of the Network Maturity Grid model (Systems of Leadership, Governance and Management, Quality Improvement, Engagement and Community Building, Data and Analytics, and Research.)	Learning network leaders**	Not reported	Informed Evaluation Tool development
National Paediatric Cardiology Quality Improvement Collaborative	Across the six domains of the Network Maturity Grid model (Systems of Leadership, Governance and Management, Quality Improvement, Engagement and Community Building, Data and Analytics, and Research.)	Learning network leaders**	Not reported	Informed Evaluation Tool development and As Strategic Planning Tool
Autism Speaks: Autism Treatment Network/Autism Intervention Research Network on Physical Health	Across the six domains of the Network Maturity Grid model (Systems of Leadership, Governance and Management, Quality Improvement, Engagement and Community Building, Data and Analytics, and Research.)	Learning network leaders**	Not reported	Informed Evaluation Tool development
Cystic Fibrosis Learning Network	Across the six domains of the Network Maturity Grid model (Systems of Leadership, Governance and Management, Quality Improvement, Engagement and Community Building, Data and Analytics, and Research.)	Learning network leaders**	Not reported	Informed Evaluation Tool development
Improving Renal Outcomes Collaborative	Across the six domains of the Network Maturity Grid model (Systems of Leadership, Governance and Management, Quality Improvement, Engagement and Community Building, Data and Analytics, and Research.)	Learning network leaders**	Not reported	Informed Evaluation Tool development
All Children Thrive Learning Network	Across the six domains of the Network Maturity Grid model (Systems of Leadership, Governance and Management, Quality Improvement, Engagement and Community Building, Data and Analytics, and Research.)	Executive level leadership (consisting of clinicians and parents) and front-line staff (project management and quality improvement)	Not reported	Informed Evaluation Tool development
Cerebral Palsy Research Network	Across the six domains of the Network Maturity Grid model (Systems of Leadership, Governance and Management, Quality Improvement, Engagement and Community Building, Data and Analytics, and Research.)	Systems level leaders (*n* = 10), network research principal investigators (*n* = 5), and Quality Improvement Project Leaders (*n* = 4)	Not reported	As Strategic Planning Tool

**No further detail provided

Notably, eight out of nine of the examples were conducted during the initial development of the NMG and only one case study (Cerebral Palsy Research Network) applied the tool in its final format. [4].

Stakeholders at a leadership level were approached as participants in the evaluation process in all identified case studies, with a focus on executive leadership. However, detailed breakdowns of different leadership levels were only available for one case study, specifically the evaluation of the Cerebral Palsy Research Network [4]. In this instance, stakeholders at systems leadership (*n* = 10), academic leadership (*n* = 5), and quality improvement project leadership (*n* = 4) levels were approached, with a resulting 68% (*n* = 13) response rate.

One example noted a potential use of the results of evaluation as a strategic planning tool to direct future investment of resources and improvement [20] during initial development. This was further corroborated by the Cerebral Palsy research network case study [23], where the results of the evaluation were visualized as a radar chart, before being shared back to the survey population and used to structure a priority setting exercise to identify the top six focus areas for improvement for the future.

Additionally, none of the case studies reported end LHS outcomes as part of implementing the LHS.

## Discussion

### Summary of findings

Despite the emerging evidence supporting LHS approaches to health system improvement [2], the LHS as a theoretical framework with variable constructs can be challenging to implement in complex health systems [12]. Hence, ongoing efforts to pragmatically implement and evaluate LHS application is vital to advance the field. However, this remains an under explored area of investigation. This scoping review found three candidate tools for LHS implementation evaluation [20–22] that met the inclusion criteria. Only one (the NMG [20]) had been developed through a combination of stakeholder, theory, and implementation-based evidence. Nonetheless, the LHS constructs captured across the tools showed consistency and aligned to the structure of the Monash LHS Framework. Additionally, there was diversity in terms of evaluation methodology that was employed by these tools. The review found a further nine examples of implementation evaluation, of which eight were conducted as part of the development process of the NMG tool [20]. The remaining tool applied the NMG tool post development [23]. The examples constitute nine different clinical areas, all at a regional level, with evaluation been conducted with participants at different leadership levels. As a result, the NMG tool emerged as the most mature implementation evaluation tool that was identified, suitable for further adaptation and iteration. As a result, the NMG tool is being adapted and iterated upon as part of ongoing work on the Monash LHS model within Australian government funded programs.

### Identified tools converged in terms of evaluated constructs

A convergence in the evaluated constructs that was observed across all the three identified tools (Table 2), particularly between the Network Maturity Grid and the KPWA LHS Logic Model, affirming a shared understanding of the core constructs that constitute a LHS. These five constructs are: stakeholders, research evidence, data, implementation, and sociotechnical infrastructure, as detailed in Table 2, and also align with the four evidence quadrants (stakeholders, research evidence, practice evidence/data, and implementation) in the Monash LHS Framework (Fig. 1).

Additionally, both the NMG [20] and KPWA LHS Logic Model [21] take an implicit process-as-outcome lens, highlighting key activities that would constitute a functional LHS and, in theory, achieve better outcomes. As such, they enable practical application of the erstwhile theoretical nature of LHSs as a pragmatic set of processes across the five core constructs that can be practically implemented, evaluated, and iteratively improved across various stages of maturity and fidelity.

### Identified tools diverged in terms of evaluation methodology

Notably, there was also divergence in the evaluation methodologies that were employed by the identified tools. From the NMG’s numerical rating to the LADDERS tool’s form-based prompts and the qualitative depth of the KPWA LHS Logic Model. Such divergence indicates that while the content of what should be evaluated when it comes to LHS implementation should be standardized, the format and application of these tools require customization to the context in which they are deployed. Consequently, the development and implementation of an LHS evaluation toolkit may require a dual approach: standardization in “what” is measured to maintain comprehensive and comparable evaluations, alongside flexibility in how measurements and evaluation are conducted to accommodate the unique environments of various LHSs. Such tools may also need to consider the burden of data collection to facilitate evaluation, and alternative approaches to toolkit implementation may be required, such as integration into existing data infrastructure [24] or the use of researchers in residence [25] to facilitate qualitative evaluations.

### Learnings from implementation evaluation examples

Encouragingly, all included examples differed in terms of clinical area, including, but not limited to, perinatal health, cerebral palsy, cardiology, and autism; demonstrating the flexibility of the application of the LHS as a framework: and highlighting that evaluation can be carried out in a field-agnostic manner. Further, the included examples [20, 23] indicate that evaluation should be carried at various levels of leadership, starting at project level, to health service level, and to executive and system level. However, while leadership buy-in is important, this top-down approach should be in addition to a bottom-up approach to evaluation [26] that captures community-generated interventions and the latent knowledge of end users, beneficiaries, and workers on the front line. Indeed, such combined approaches [27] have been shown to support both the implementation [28, 29] and deimplementation [30] of complex interventions within healthcare settings allowing flexibility in how such an evaluation toolkit will be implemented, while maintaining consistency in constructs being evaluated. Lastly, the examples indicate that results of evaluation should be framed as a both (1) a way to track progress in realizing a LHS and (2) prioritize areas for process and infrastructure improvement and later investment.

### Implications for implementation evaluation toolkit development and implementation

Collectively, our results suggest that an effective LHS implementation evaluation toolkit needs to first consistently evaluate an LHS exemplar as an integrated suite of interventions across all five key constructs (stakeholders, research evidence, data, implementation, and sociotechnical Infrastructure). This should be framed around assessing how well the existing implementation or fidelity [31] of a LHS aligns with the LHS as an aspirational model. Second, the LHS implementation evaluation toolkit should also have mechanisms to evaluate how well the LHS exemplar at a given fidelity has successfully achieved its stated aims, for example, in improving stroke outcomes. This, thus, provides a concrete link between the LHS as an aspirational vision, to the LHS in practice, to outcomes that matter to stakeholders. Such an approach would help delineate whether failure or success may be attributable toward the LHS approach itself or issues with its implementation and has been shown to be helpful in tailoring complex interventions for further scale up [32, 33].

### Limitations

The search for this scoping review was conducted only on peer-reviewed literature, and it is possible that the gray literature would yield additional case studies of LHS implementation evaluation. Further, all of the implementation evaluation examples and tools identified were in the USA within regional scale health systems, limiting generalizability beyond this environment, particularly in low resource contexts. Additionally, none of the implementation evaluation examples reported health outcomes as a direct result of LHS implementation, and none of the tools identified an approach to systematically identify stakeholders and engage them as a part of LHS evaluation toolkit development and subsequent evaluation, limiting learning applicable for further toolkit development efforts. Lastly, the search considered contexts that self-identified as an LHS, and it is likely that other environments that are LHS-like may not have been captured in this review.

## Conclusions

The learning health system presents a proven approach toward health system improvement that has shown considerable promise in generating cyclical and measurable healthcare improvement. However, much of the literature remains theoretical, and our scoping review has captured emerging empirical evidence to both guide the implementation of the LHS in the first instance and guide the evaluation of LHS implementation to ensure consistent alignment with the aspirational vision of the LHS. We have shown consistency in the LHS constructs that should be measured by these tools and a divergence in how such evaluations can be implemented. As such, further iteration and adaptation of LHS evaluation toolkits should consider evaluation across five core constructs (evidence from stakeholders, research, practice/data, and implementation, as well as sociotechnical infrastructure), while maintaining flexibility in evaluation methodology to allow for adaptation to local contexts. Further, more work is needed in evaluating the use of these tools across diverse clinical settings beyond the USA, across low and middle income settings, and underserved populations, and settings including regional and rural care with further focus on linking the implementation of the LHS with direct health system outcomes.

## Data Availability

All data supporting the findings of this study is available within the paper and the accompanying supplementary information.

## Appendix 1

### Search code used on the electronic databases

A systematic search, based on the selection criteria and combining key words, was developed. The search strategy is limited to English language papers published in the previous five years.

Medline: (learn* health* system*.tw) OR (learn* health* network*.tw) OR (learn* network*.tw) OR (learn* org*.tw) AND (evaluat* or assess* or matur* or implem*).tw. AND (limit to (yr="2018-Current" and English))

Medline in-process (and other non-indexed citations):

Same as for Medline

Embase:

Same as for Medline

Scopus: ( TITLE-ABS-KEY ( “learning health system*”) OR (“learning health network*”) OR (“learning network*”) AND ( health* OR ehealth ) AND ( partner* OR collaborat* OR "startup" ) AND ( data* OR informatic* OR infomatic* OR digital ) AND ( translation* ) AND (evaluat* OR assess* OR matur*) AND ( LIMIT-TO ( PUBYEAR , 2023 ) OR LIMIT-TO ( PUBYEAR , 2022 ) OR LIMIT-TO ( PUBYEAR , 2021 ) OR LIMIT-TO ( PUBYEAR , 2020 ) OR LIMIT-TO ( PUBYEAR , 2019 ) OR LIMIT-TO ( PUBYEAR , 2018 ) AND ( LIMIT-TO ( LANGUAGE , "English" ) )
